# Clinical activity of chidamide-containing regimens after prior PD-1-based therapy in two patients with microsatellite-stable metastatic colorectal cancer: a case series and narrative review

**DOI:** 10.3389/fonc.2026.1905769

**Published:** 2026-09-08

**Authors:** Runzhi Chen, Dongmei Yang, Huiting Xu

**Affiliations:** 1 Department of Medical Oncology, Huazhong University of Science and Technology, Tongji Medical College, Hubei Cancer Hospital, Wuhan, Hubei, China; 2 Hubei Provincial Clinical Research Center for Colorectal Cancer, Wuhan, Hubei, China; 3 Wuhan Clinical Research Center for Colorectal Cancer, Wuhan, Hubei, China

**Keywords:** colorectal cancer, epigenetic therapy, HDAC inhibitor, immunotherapy resistance, microsatellite stable

## Abstract

**Background:**

More than 90% of metastatic colorectal cancers (mCRC) are microsatellite-stable (MSS), an immunologically “cold” subtype characterized by low neoantigen burden, impaired antigen presentation, and poor responsiveness to immune checkpoint inhibitors (ICIs). Epigenetic aberrations—including aberrant DNA methylation and histone deacetylation—drive immune evasion in MSS CRC. Histone deacetylase inhibitors (HDACi) can restore major histocompatibility complex class I (MHC-I) expression, reshape the immunosuppressive tumor microenvironment (TME), and synergize with ICIs to enhance antitumor immunity. This retrospective study evaluated chidamide (a selective HDACi) plus a PD-1 inhibitor in two MSS CRC patients with prior ICI progression, with a mechanistic discussion informed by published evidence.

**Case presentation:**

Case 1: A 60-year-old woman with BRAF V600E-mutant, right-sided, MSS mCRC received third-line surufatinib plus camrelizumab, with a best response of stable disease (SD) and a progression-free survival (PFS) of 5 months. After subsequent disease progression, fifth-line chidamide plus sintilimab achieved a partial response (PR), with a PFS of 6 months. Case 2: A 43-year-old man with recurrent RAS/BRAF wild-type MSS mCRC received fifth-line surufatinib plus sintilimab (PR, PFS 8.5 months) and, after progression, seventh-line chidamide plus sintilimab plus bevacizumab (SD, PFS 5.5 months). No grade 3–4 AEs occurred.

**Conclusion:**

This retrospective two-patient case series observed one PR and one SD in heavily pretreated MSS mCRC patients with prior ICI failure who received chidamide plus a PD-1 inhibitor (Case 1: primary resistance; Case 2: acquired resistance). As descriptive observations, these findings are hypothesis-generating only and are insufficient to demonstrate reversal of immunotherapy resistance or survival benefit. Prospective biomarker-driven studies are warranted to evaluate epigenetic-immunotherapy combinations in this subgroup.

## 1 Introduction

Colorectal cancer (CRC) is one of the most prevalent malignancies worldwide and a leading cause of cancer-related mortality. According to GLOBOCAN 2022 estimates, there were approximately 1.9 million new CRC cases and 900,000 related deaths globally, making it the third most common cancer and the second leading cause of cancer death. The global disease burden is projected to continue to increase, with an estimated 3.2 million new cases and 1.6 million deaths per year by 2040, alongside a rising incidence in young adults (1). Roughly 25% of patients present with metastatic CRC (mCRC) at initial diagnosis, of whom over 90% have the microsatellite-stable (MSS) subtype, which corresponds to proficient mismatch repair (pMMR) by immunohistochemistry (IHC) in routine practice (2, 3).

Chemotherapy combined with anti-EGFR or anti-VEGF monoclonal antibodies remains the standard first-line treatment for mCRC, with a median overall survival (OS) of approximately 30 months. However, long-term outcomes remain unsatisfactory (4, 5). Over the past decade, immune checkpoint inhibitors (ICIs) have revolutionized the treatment landscape of multiple solid tumors. However, unlike microsatellite instability-high (MSI-H) CRC, which derives substantial benefit from ICI therapy, MSS tumors are immunologically “cold” and poorly responsive to ICIs. Low tumor mutational burden (TMB), insufficient neoantigen production, and impaired antigen presentation jointly hinder the activation of tumor-specific CD8^+^ T cells, resulting in inherent resistance to ICIs (6). Therefore, developing novel strategies to convert immunologically “cold” MSS tumors into “hot” tumors represents an unmet clinical need.

Current therapeutic strategies for MSS mCRC predominantly focus on combination regimens, including ICIs plus chemotherapy, targeted agents, or radiotherapy. The REGONIVO trial reported that regorafenib plus nivolumab yielded an objective response rate (ORR) of 36%, whereas the overall ORR of anti-angiogenic drugs combined with PD-1 inhibitors was approximately 15% in real-world settings (7, 8). Novel therapeutic modalities such as bispecific antibodies, neoantigen vaccines, and adoptive cell therapy are also being evaluated in early-phase trials, although their efficacy and safety warrant further validation (9).

Accumulating evidence has demonstrated that epigenetic regulation plays a critical role in tumor immune evasion. Histone deacetylase inhibitors (HDACi) and DNA methyltransferase inhibitors have been shown to upregulate MHC-I expression, remodel the tumor microenvironment (TME), and restore tumor cell sensitivity to ICIs (10–12). Multiple preclinical studies and phase II clinical trials have suggested the synergistic effect of epigenetic drugs and immunotherapy (13–15). Multiple preclinical studies and phase II clinical trials have suggested synergistic activity of epigenetic agents combined with immunotherapy. However, most existing clinical studies have enrolled ICI-naive patients, and data on epigenetic agents in MSS mCRC after prior ICI progression are limited.

Herein, we report two heavily pretreated MSS mCRC patients who achieved temporary radiological disease control after switching to chidamide plus a PD-1 inhibitor following prior ICI progression. We also summarize the mechanisms of immune resistance in MSS CRC and recent advances in epigenetic–immunotherapy combinations, which may help inform the management of ICI-refractory MSS mCRC.

## 2 Case presentations

All treatment responses were evaluated according to the Response Evaluation Criteria in Solid Tumors (RECIST) version 1.1. Adverse events (AEs) were assessed using the Common Terminology Criteria for Adverse Events (CTCAE) version 5.0. Immunotherapy resistance was defined according to the Society for Immunotherapy of Cancer (SITC) consensus criteria (16): primary resistance was defined as progressive disease (PD) as the best response or PD within 6 months of ICI initiation; acquired resistance was defined as PD after an initial objective response (CR or PR) or after stable disease (SD) lasting ≥6 months on ICI-based therapy. All patient identifiers have been anonymized **Tables 1**, **2**.

**Table 1:** Patient characteristics of the two cases.

Characteristic	Case 1	Case 2
Demographics		
Age/Sex	60/F	43/M
ECOG	0	0
Primary tumor site	Right colon	Upper rectum
Histology	Moderately-to-poorly differentiated adenocarcinoma	Adenocarcinoma
Pathological/Molecular profile	pT4aN2b, pMMR, BRAF V600E (+), KRAS/NRAS WT	cT4N+M1, pMMR, KRAS/NRAS/BRAF WT
Metastatic sites	Peritoneal, lymph nodes, lungs	Liver, lymph nodes
TMB	NA	NA
POLE/POLD1	NA	NA
PD-L1	NA	NA

ECOG, Eastern Cooperative Oncology Group; pMMR, Proficient Mismatch Repair; WT, wild-type; NA, not available.

**Table 2:** Sequential treatment history and best responses of the two cases.

Line	Regimen	Treatment period	Best response	PFS (moths)
Case 1				
1st	FOLFOXIRI + bevacizumab	Aug–Dec 2022	PD	5
2nd	Cetuximab+dabrafenib+trametinib	Dec 2022–Jul 2023	PR	6
3rd	Camrelizumab+ surufatinib	Jul 2023–Dec 2023	SD→SD→PD	5
4th	Irinotecan + cetuximab+ vemurafenib	Dec 2023–Feb 2024	PD	2
5th	Sintilimab+ chidamide	Feb–Aug 2024	PR	6
6th	Bevacizumab + TAS-102	Aug 2024–Jan 2025	SD→PD	4
7th	Regorafenib + TAS-102+adrenal radiation therapy	Jan 2025–Jun 2025	SD	5
Case 2				
1st	Cetuximab + FOLFOX6	Jun–Dec 2021	PR→PD	6
2nd	Bevacizumab + FOLFIRI → bevacizumab + capecitabine	Dec 2021–Oct 2022	SD→PD	11
3rd	Cetuximab + irinotecan	Oct 2022–Mar 2023	SD→PD	5
4th	Regorafenib	Mar–Apr 2023	PD	1
5th	Sintilimab + surufatinib	Apr 2023–Jan 2024	SD→PR→PD	8.5
6th	Bevacizumab + TAS-102	Jan–Apr 2024	PD	2.5
7th	Sintilimab + bevacizumab + chidamide	Apr–Sep 2024	SD	5.5
8th	Cetuximab + oxaliplatin + irinotecan + raltitrexed	Sep 2024–Mar 2025	PR→PD	4.5
9th	Lenvatinib + S-1	Mar–May 2025	PD	2

All responses and progressions were assessed per RECIST 1.1 criteria; pMMR status was confirmed by IHC for both cases; PFS, progression-free survival; PR, partial response; SD, stable disease; PD, progressive disease.

### 2.1 Case 1

A 60-year-old Chinese female patient was diagnosed with moderately/poorly differentiated right-sided colon adenocarcinoma (pT4aN2b) in July 2022. Postoperative pathology revealed lymphovascular invasion, lymph node metastasis (20/31), and multiple distant tumor deposits. IHC confirmed pMMR, and molecular testing identified BRAF V600E mutation with KRAS/NRAS wild-type.

**Figure 1:**
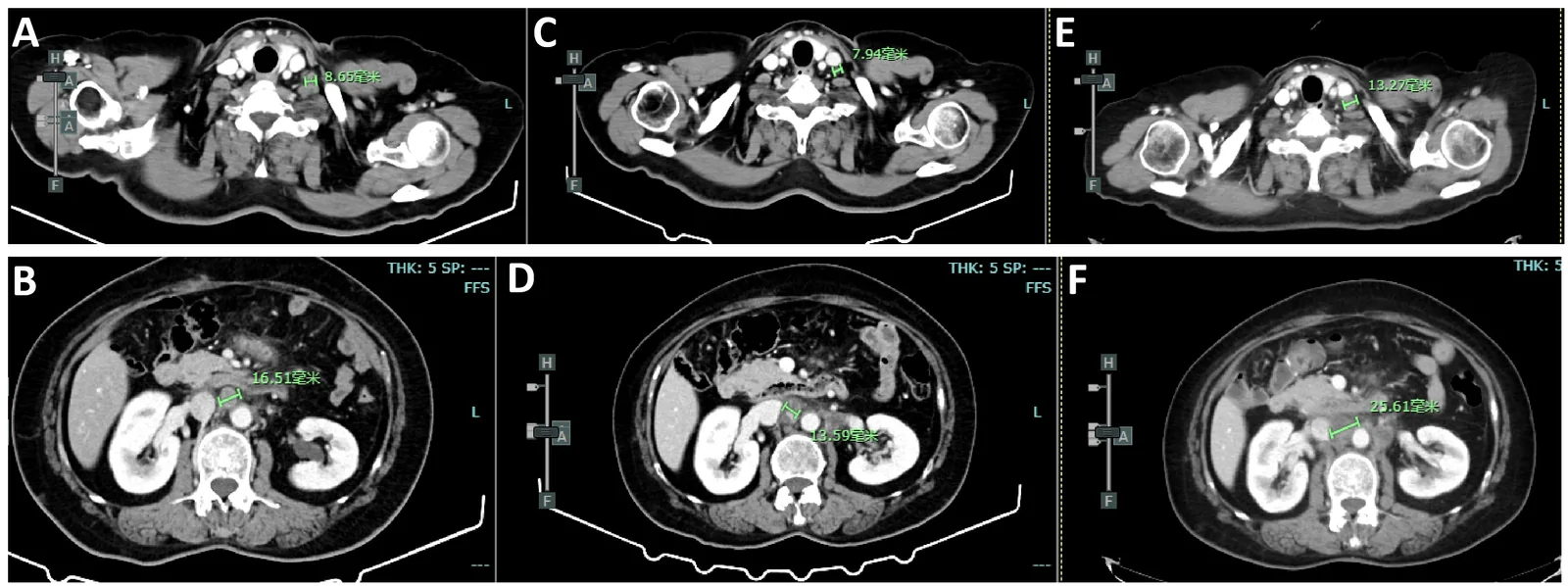
Serial chest CT images during third-line camrelizumab plus surufatinib therapy. **(A, D)** Baseline (July 1, 2023). Target lesion: 1.6 cm. **(B, E)** Best response (October 2, 2023). Target lesion reduced to 1.3 cm (−18.8%); per RECIST, best overall response: stable disease (SD). **(C, F)** Progression (December 18, 2023). Target lesion enlarged to 2.6 cm (+62.5%) with new/progressive non-target lesions; per RECIST, progressive disease (PD). Scan dates are shown in each panel. All images are at comparable anatomical levels.

The patient received first-line FOLFOXIRI plus bevacizumab and second-line dabrafenib plus trametinib plus cetuximab, with disease progression occurring after each regimen. Her third-line treatment was surufatinib (250 mg orally once daily) plus camrelizumab (200 mg intravenously every three weeks). The best response per RECIST was SD, with the target lesion reducing from 1.6 cm at baseline to 1.3 cm at best response. Disease progression occurred after 5 months of treatment (PFS = 5 months), as shown in **Figure 1**.

**Figure 2:**
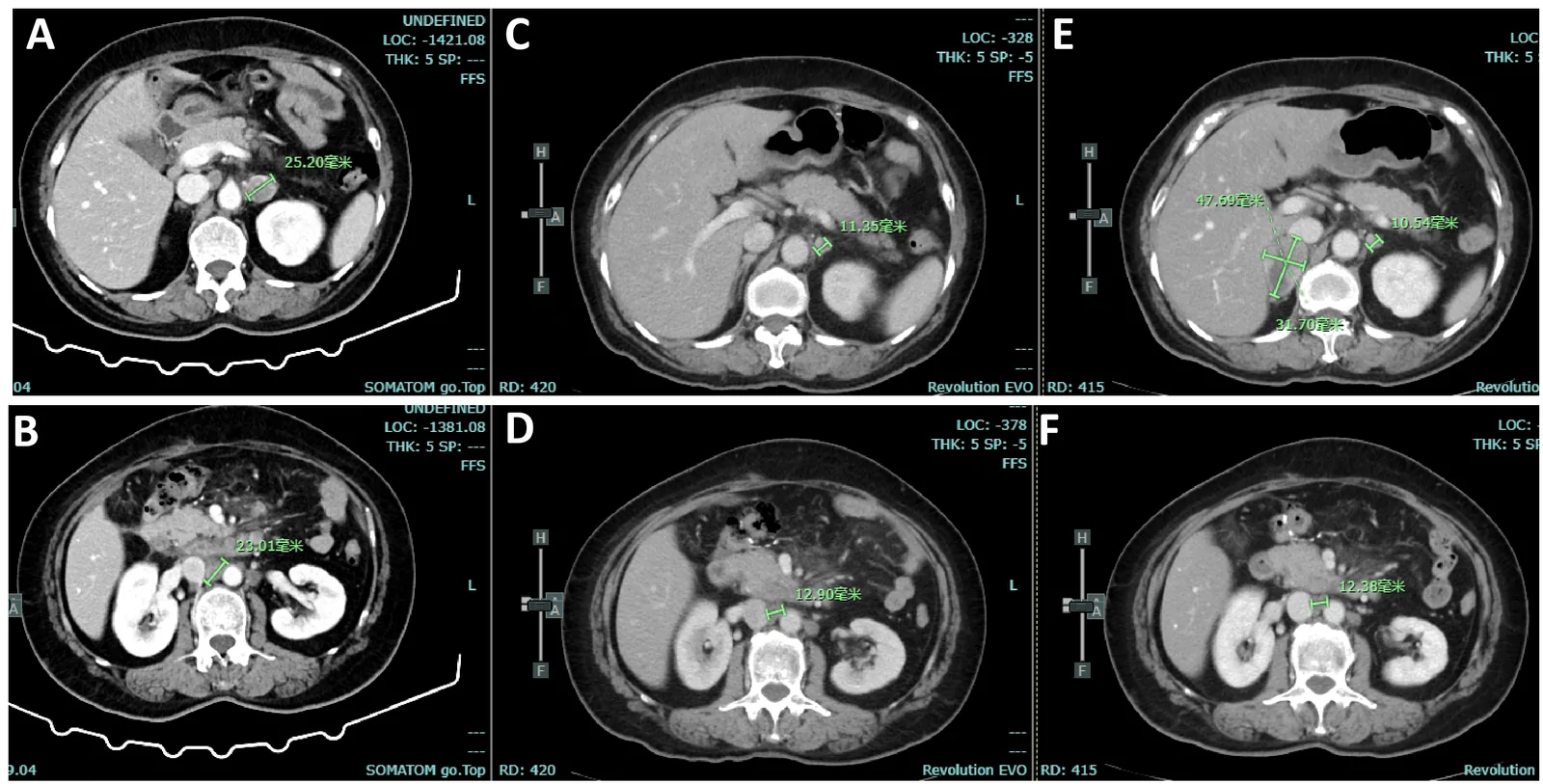
Serial CT images during fifth-line sintilimab plus chidamide therapy. **(A, D)** Baseline (February 21, 2024). Sum of target lesions: 4.8 cm. **(B, E)** Best response (May 27, 2024). Sum of target lesions reduced to 2.3 cm (−52.1%); per RECIST, best overall response: partial response (PR). **(C, F)** Progression (August 26, 2024). Target lesions stable, with new right adrenal lesion; per RECIST, progressive disease (PD). Scan dates are shown in each panel. All images are at comparable anatomical levels.

**Figure 3:**
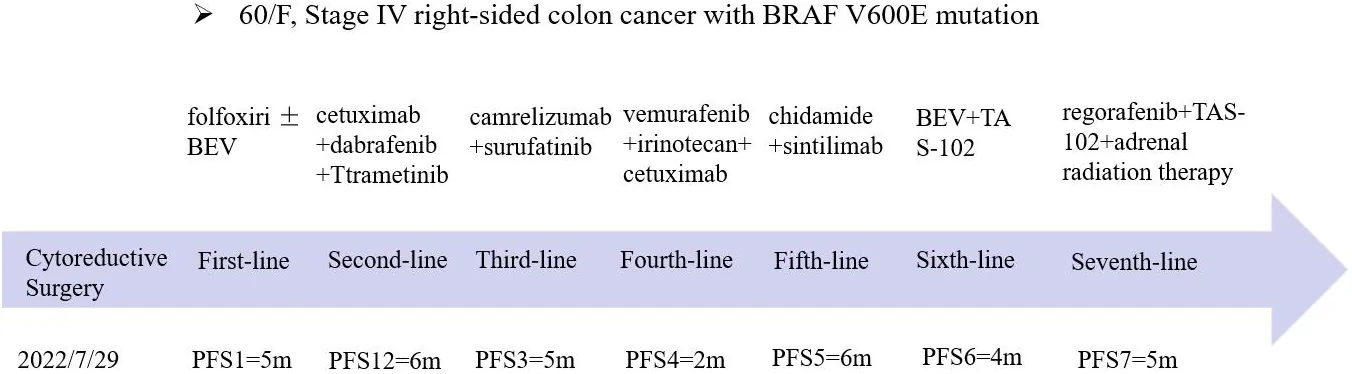
Timeline of the case-1 patient’s treatment history.

Fourth-line irinotecan plus cetuximab plus vemurafenib also led to early treatment failure, with a PFS of 2 months. In February 2024, the patient received fifth-line chidamide (20 mg orally twice weekly) plus sintilimab (200 mg IV every 3 weeks) for 7 cycles. Best response was PR (target lesions: 4.8 cm → 2.3 cm; PFS 6 months), followed by PD with new right adrenal lesions (**Figure 2**). Grade 2 hepatic enzyme abnormalities developed during treatment and resolved with symptomatic management; no other AEs were observed. After progression, the patient received bevacizumab plus trifluridine/tipiracil and regorafenib sequentially. The detailed treatment timeline is shown in **Figure 3**. The last follow-up was in June 2025.

### 2.2 Case 2

A 43-year-old Chinese male patient was diagnosed with stage IV rectal adenocarcinoma (cT4N1M1) with liver and multiple lymph node metastases in June 2021. IHC confirmed pMMR, and molecular testing showed RAS/BRAF wild-type.

**Figure 4:**
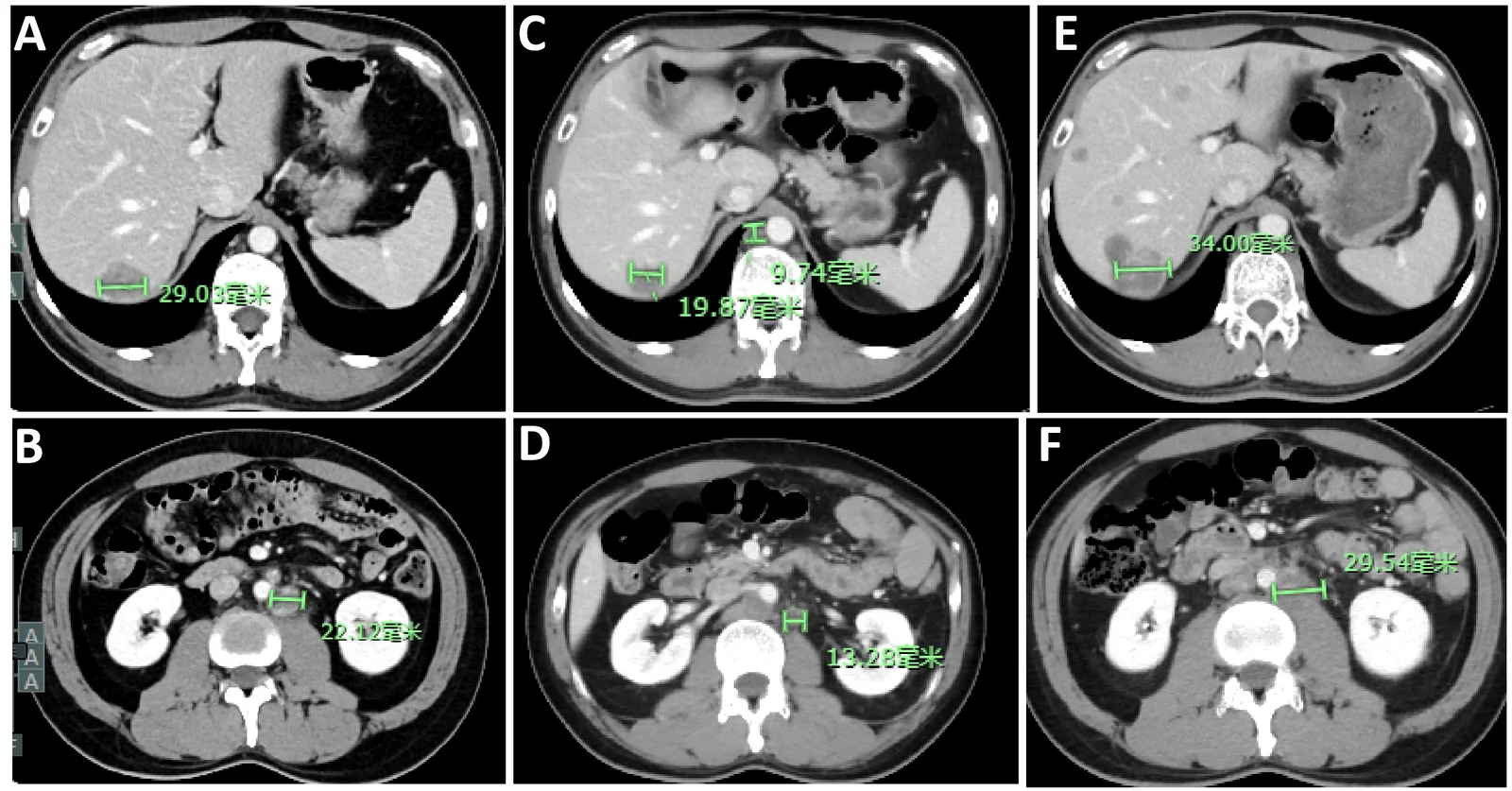
Serial CT images during fifth-line sintilimab plus surufatinib therapy. **(A, C)** Baseline (April 14, 2023). Sum of target lesions: 5.1 cm. **(B, D)** Best response (October 11, 2023). Sum of target lesions reduced to 2.2 cm (−56.9%); per RECIST, best overall response: partial response (PR). **(E, F)** Progression (January 24, 2024). Target lesions enlarged to 6.3 cm (+23.5% vs. baseline); per RECIST, progressive disease (PD). Scan dates are shown in each panel. All images are at comparable anatomical levels.

**Figure 5:**
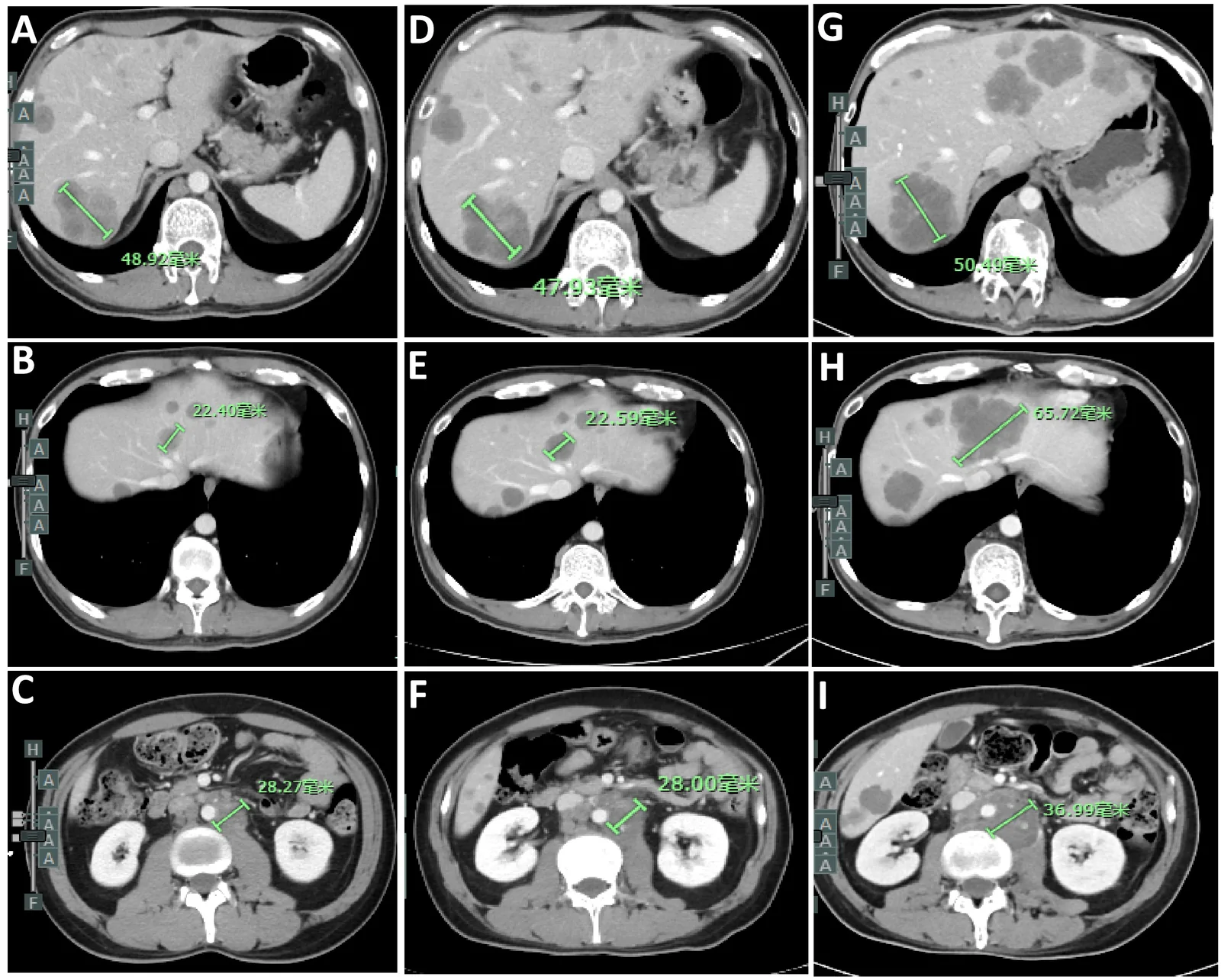
Serial CT images during seventh-line bevacizumab plus sintilimab and chidamide therapy. **(A–C)** Baseline (April 1, 2024). Sum of target lesions: 9.9 cm. **(D–F)** Best response (May 16, 2024). Sum of target lesions reduced to 9.8 cm (−1.0%); per RECIST, best overall response: stable disease (SD). **(G–I)** Progression (September 24, 2024). Sum of target lesions enlarged to 15.0 cm (+51.5% vs. baseline); per RECIST, progressive disease (PD). Scan dates are shown in each panel. All images are at comparable anatomical levels.

**Figure 6:**
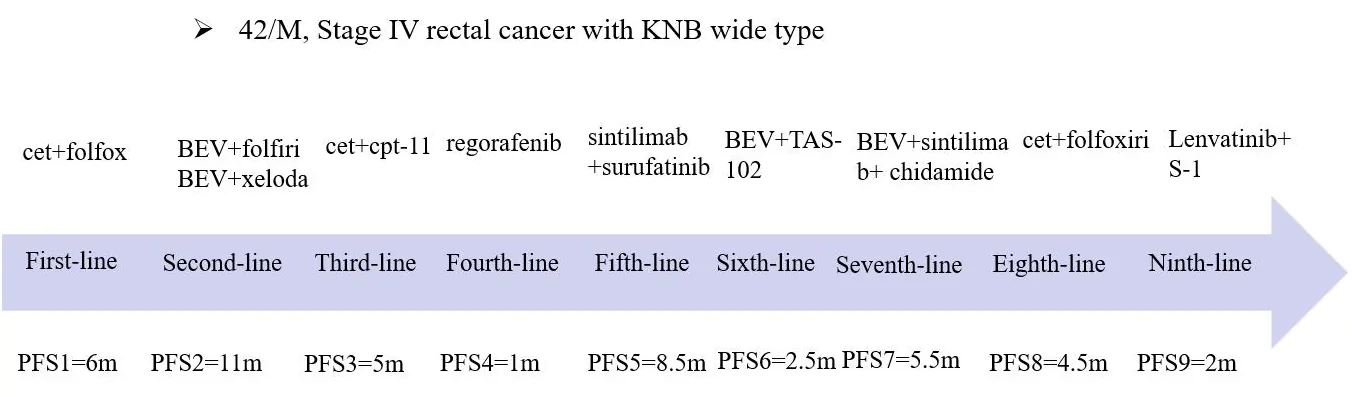
Timeline of the case-2 patient’s treatment history.

The patient progressed after multiple lines of chemotherapy and targeted therapy. Her fifth-line treatment was surufatinib (250 mg orally once daily) plus sintilimab (200 mg intravenously every three weeks). The best response per RECIST was a PR, with the target lesion decreasing from 5.1 cm at baseline to 2.2 cm at best response. Disease progression occurred after 8.5 months (PFS = 8.5 months), as shown in **Figure 4**. After failure of sixth-line bevacizumab plus trifluridine/tipiracil (PFS: 2.5 months), he received seventh-line chidamide (20 mg orally twice weekly), sintilimab (200 mg IV every 3 weeks), and bevacizumab (15 mg/kg IV every 3 weeks) for 7 cycles. The target lesion showed a minimal decrease from 9.9 cm to 9.8 cm, and SD was maintained for 5.5 months (PFS: 5.5 months), as shown in **Figure 5**. Grade 1 hypothyroidism developed during treatment; no other AEs were observed. The detailed treatment timeline is shown in **Figure 6**. The last follow-up was in May 2025.

## 3 Discussion

### 3.1 Epigenetic therapy remodels anti-tumor immunity: preclinical rationale

Epigenetic modifications refer to heritable changes in gene expression without alterations to DNA sequences, including DNA methylation, histone acetylation/methylation, RNA modification, and chromatin remodeling (17–20). Epigenetic dysregulation is a core driver of tumor immune evasion in MSS CRC, and epigenetic drugs have been investigated as a strategy to modulate immune resistance in this subtype of CRC. HDACi and DNA methyltransferase inhibitors exert immune-modulatory effects through multiple pathways in preclinical models.

#### 3.1.1 Restoring antigen presentation

Aberrant histone deacetylation and DNA methylation can silence MHC-I antigen processing genes, including MHC-I, B2M (beta-2 microglobulin), TAP1 (transporter associated with antigen processing 1), and TAP2 (21), contributing to poor ICI responsiveness in MSS CRC. Both HDACi and DNMTis (DNA methyltransferase inhibitors) can reverse this epigenetic repression and restore MHC-I expression (21), with DNMTis specifically shown to upregulate neoantigen-bearing gene expression and enhance neoantigen-specific T-cell cytotoxicity in MSS CRC models (22).

#### 3.1.2 Activation of innate immune sensing

Demethylating agents can reactivate endogenous retroviruses (ERVs), triggering dsRNA-mediated interferon responses via the MDA5/MAVS pathway and downstream IRF7 activation, converting immune-cold tumors into immune-hot tumors in colorectal cancer models (23). STING activation has also been implicated in epigenetic therapy-induced antitumor immunity, and synthetic STING agonists have shown systemic efficacy in colon tumor models (24), suggesting alternative targets along the innate immune axis.

#### 3.1.3 Modulating immune cell function

In the immunosuppressive TME of MSS CRC, epigenetic drugs can regulate immune cell function by enhancing DCs (dendritic cells) and influencing CD4^+^ T-cell differentiation (25, 26). Chronic stimulation drives CD8^+^ T-cell exhaustion with a fixed epigenetic scar that persists even after antigen clearance (27, 28). Epigenetic modulators may thus remodel this exhausted chromatin landscape to reinvigorate T cells, providing a rationale for their combination with ICIs in MSS CRC.

Collectively, HDACi and DNMTi reshape the TME via antigen presentation restoration, viral mimicry, immune cell modulation, and T-cell reinvigoration (29). Preclinical data support synergy with ICIs, though clinical evidence in MSS CRC is limited (14). The dual DNMT/HDACi C02S has shown immune-dependent antitumor activity in CRC models, increasing T/NK cell infiltration and reducing immunosuppressive populations (30). These findings support further clinical investigation of epigenetic–immunotherapy combinations in MSS CRC.

### 3.2 Clinical progress of epigenetic combined immunotherapy for MSS CRC

A series of phase II clinical trials has suggested the potential efficacy of epigenetic agents plus ICIs in MSS mCRC. The MAYA trial showed that temozolomide combined with low-dose ipilimumab and nivolumab achieved an ORR of 30% and a median OS of 12.5 months in MGMT-methylated MSS mCRC patients (13). In another phase II study of MSS-type advanced CRC patients who progressed after ≥1 prior therapy, the triplet combination of azacitidine, romidepsin, and pembrolizumab showed modest activity, with one durable PR lasting 19 months (14). The CAPability-01 trial provided randomized phase II evidence for activity of a chidamide-containing triplet regimen in selected ICI-naive patients with MSS (pMMR) mCRC (15).

Most prior studies enrolled ICI-naive patients. The interpretation of findings in previously ICI-exposed populations requires separate consideration, as the biological context and expected response patterns may differ substantially from those in ICI-naive settings.

### 3.3 Interpretation of the present observations

Several preclinical and early clinical studies have specifically investigated HDACi–ICI combinations after prior ICI exposure. In colon cancer models, chidamide combined with TKIs (tyrosine kinase inhibitors) and ICIs remodeled the TME by reducing immunosuppressive cells and restoring T-cell activation in ICI-resistant tumors (31). In patients with classic Hodgkin lymphoma (cHL) refractory to prior epigenetic–immunotherapy, the chidamide-containing CDP regimen (chidamide, decitabine, and a PD-1 inhibitor) achieved high response rates, with correlative studies suggesting CD8^+^ T-cell activation and STAT1/3 pathway modulation (32). However, extrapolation from hematological malignancies to MSS CRC requires substantial caution, and the applicability of these findings to the ICI-exposed, immunotherapy-resistant solid tumor setting remains uncertain.

In this context, we describe two cases of heavily pretreated MSS mCRC patients with documented disease progression on prior PD-1 inhibitor-containing therapy who subsequently received chidamide-containing regimens and achieved radiological disease control. Nevertheless, the two cases differed substantially in their prior ICI resistance patterns (Case 1: primary resistance; Case 2: acquired resistance) and in the subsequent regimens—both received an ICI-containing regimen after an interval without ICI therapy, but Case 1 switched to a different PD-1 inhibitor combined with chidamide, whereas Case 2 resumed the same PD-1 inhibitor combined with chidamide and bevacizumab. These differences preclude direct comparison and complicate attribution of the observed disease control to chidamide alone. Despite distinct molecular backgrounds (BRAF V600E-mutant vs. RAS/BRAF wild-type), both patients achieved temporary radiological disease control, raising the hypothesis that chidamide-containing regimens might have activity beyond a single molecular subset—a hypothesis that requires prospective validation.

### 3.4 Limitations and future perspectives

Several limitations should be acknowledged. Methodologically, the retrospective two-case series with no control group precludes causal attribution and generalizability. Procedurally, the use of different combination regimens across cases limits isolation of individual drug effects. Definitionally, the two cases represent distinct resistance categories (primary vs. acquired). Mechanistically, the absence of on-treatment biospecimens prevents elucidation of the underlying biological mechanisms that might account for the observed disease control. Externally, the single-center design and potential publication bias constrain broader applicability. These findings are therefore hypothesis-generating, not conclusive.

Future efforts should prioritize prospective controlled trials to validate efficacy and optimize dosing/combination strategies, alongside biomarker-driven studies to enable rational patient selection. Multidimensional combination approaches integrating epigenetic, immune, and metabolic targets also warrant further exploration in refractory MSS CRC.

## 4 Conclusion

These two cases describe radiological disease control with chidamide-containing regimens after prior PD-1-based therapy in heavily pretreated MSS metastatic colorectal cancer. Because of regimen heterogeneity, concomitant anti-VEGF therapy, intervening treatments, and the absence of mechanistic biomarkers, these observations cannot establish reversal of acquired immunotherapy resistance. Prospective studies with predefined resistance criteria and paired translational analyses are required.
